# Platelet Mitochondrial Bioenergetics Reprogramming in Patients with Urothelial Carcinoma

**DOI:** 10.3390/ijms23010388

**Published:** 2021-12-30

**Authors:** Patrik Palacka, Anna Gvozdjáková, Zuzana Rausová, Jarmila Kucharská, Ján Slopovský, Jana Obertová, Daniel Furka, Samuel Furka, Keshav K. Singh, Zuzana Sumbalová

**Affiliations:** 1 2nd Department of Oncology, Faculty of Medicine, Comenius University in Bratislava, Klenova 1, 833 10 Bratislava, Slovakia; jan.slopovsky@nou.sk (J.S.); jana.obertova@nou.sk (J.O.); 2 National Cancer Institute, 833 10 Bratislava, Slovakia; 3 Pharmacobiochemical Laboratory of the 3rd Department of Internal Medicine, Faculty of Medicine, Comenius University in Bratislava, 813 72 Bratislava, Slovakia; anna.gvozdjakova@fmed.uniba.sk (A.G.); zuzana.rausova@fmed.uniba.sk (Z.R.); jarmila.kucharska@fmed.uniba.sk (J.K.); zuzana.sumbalova@fmed.uniba.sk (Z.S.); 4 Department of Physical and Theoretical Chemistry, Faculty of Natural Sciences, Comenius University in Bratislava, 841 04 Bratislava, Slovakia; furka2@uniba.sk (D.F.); furka3@uniba.sk (S.F.); 5 Department of Genetics, Heersink School of Medicine, University of Alabama at Birmingham, Birmingham, AL 35294, USA; kksingh@uab.edu

**Keywords:** urothelial carcinoma, platelets, mitochondrial bioenergetics, oxidative stress, reprogramming

## Abstract

Mitochondrial bioenergetics reprogramming is an essential response of cells to stress. Platelets, an accessible source of mitochondria, have a crucial role in cancer development; however, the platelet mitochondrial function has not been studied in urothelial carcinoma (UC) patients. A total of 15 patients with UC and 15 healthy controls were included in the study. Parameters of platelet mitochondrial respiration were evaluated using the high-resolution respirometry method, and the selected antioxidant levels were determined by HPLC. In addition, oxidative stress was evaluated by the thiobarbituric acid reactive substances (TBARS) concentration in plasma. We demonstrated deficient platelet mitochondrial respiratory chain functions, oxidative phosphorylation (OXPHOS), and electron transfer (ET) capacity with complex I (CI)-linked substrates, and reduced the endogenous platelet coenzyme Q_10_ (CoQ_10_) concentration in UC patients. The activity of citrate synthase was decreased in UC patients vs. controls (*p* = 0.0191). γ-tocopherol, α-tocopherol in platelets, and β-carotene in plasma were significantly lower in UC patients (*p* = 0.0019; *p* = 0.02; *p* = 0.0387, respectively), whereas the plasma concentration of TBARS was increased (*p* = 0.0022) vs. controls. The changes in platelet mitochondrial bioenergetics are consistent with cell metabolism reprogramming in UC patients. We suppose that increased oxidative stress, decreased OXPHOS, and a reduced platelet endogenous CoQ_10_ level can contribute to the reprogramming of platelet mitochondrial OXPHOS toward the activation of glycolysis. The impaired mitochondrial function can contribute to increased oxidative stress by triggering the reverse electron transport from the CoQ_10_ cycle (Q-junction) to CI.

## 1. Introduction

Bladder cancer is the tenth most common form of cancer worldwide, with an estimated 549,000 new cases and 200,000 deaths in 2018 [1]. Malignant bladder tumors are approximately four times more common in men than in women, with incidence and mortality rates of 9.6 and 3.2 per 100,000 people, respectively. Approximately 15% of bladder cancer patients have distant metastases at the initial diagnosis [2]. Urothelial carcinoma (UC) accounts for the majority of malignant bladder tumors [3]. In approximately 25% of cases, UC differentiates into various histological subtypes; the prognosis of some variants, e.g., plasmacytoid and sarcomatoid, are worse [4]. The platelets and their role in cancer development have been in the spotlight for many years, with the first mentions dating back to the early 1970s [5,6,7]. In line with current knowledge, platelets in the blood circulation activate and encase the malignant cells detached from the primary tumor due to the secretion of growth factors and chemokines, such as vascular endothelial growth factor (VEGF), platelet-derived growth factor (PDGF), or transforming growth factor β (TGF-β). Platelet-derived growth factors also instigate tumor cell proliferation and angiogenesis to establish metastatic foci [6].

Interactions between cancer cells and activated platelets result in angiogenic regulators and microRNAs, which are delivered by platelet microparticles (MPs) to various cell types of the tumor microenvironment in favor of neovascularization [7]. Platelets mediate tumor cell arrest at the vascular wall via P-selectin and its ligands and facilitate tumor cell extravasation to the subendothelial matrix of a distant organ by activating the endothelial P_2_Y_2_ receptor. Platelets are capable of shifting the tumor cell phenotype from epithelial to mesenchymal-like [6]. Interactions between tumor cells and activated platelets upregulate epithelial–mesenchymal transition (EMT) facilitators and promote extravasation [7].

Mitochondria can trigger tumorigenesis at all stages, including initiation due to oxidative stress, signaling, and oncometabolite generated by mutations in mitochondrial enzymes; growth via metabolic reprogramming, mitochondrial signaling and biogenesis, oxidative stress, and fission/fusion dynamics; survival by metabolic reprogramming and signaling, redox homeostasis regulation, the alterations in morphology to evade the cell death, and the alterations in mitochondrial mass via the regulation of mitophagy and biogenesis; metastasis due to metabolic reprogramming, biogenesis, redox homeostasis, and fission/fusion dynamics [8,9].

Platelets isolated from peripheral blood are an accessible source of mitochondria studied in various diseases, such as lateral amyloid sclerosis [10], in patients with chronic kidney disease [11,12], in a patient after kidney transplantation [13], in patients with rheumatoid arthritis [14], and in aging [15]. Our previous study showed a reduced platelet mitochondrial metabolism and endogenous CoQ_10_ concentration in non-hospitalized patients after 3–6 weeks of acute COVID-19 disease (in platelets, CoQ_10_ was reduced vs. control to 70%, *p* = 0.002) [16].

The information on the mitochondrial bioenergetic functions of platelets in patients with cancer is not available. Therefore, we tested the hypothesis that platelet mitochondrial oxygen consumption and OXPHOS are affected in chemotherapy-naïve patients with UC.

## 2. Results

### 2.1. Blood Counts and Selected Metabolic Parameters of Subjects in Both Groups

In UC patients, both the red blood cell count and hemoglobin were significantly decreased vs. the control group (*p* < 0.0062 and *p* = 0.0014, respectively), whereas creatinine, urea, and uric acid were higher in UC patients in comparison to the control subjects (*p* < 0.0002, *p* = 0.0003 and *p* < 0.0107, respectively). On the contrary, γ-glutamyl transferase (GMT) and total bilirubin were increased in comparison with the control subjects (*p* < 0.0107 and *p* < 0.0326, respectively). The total protein and albumin were lower in patients vs. controls (*p* < 0.0042 and *p* < 0.0083, respectively) (Table 1).

### 2.2. Platelet Mitochondrial Respiration by SUIT Protocol 1

**Figure 1:**
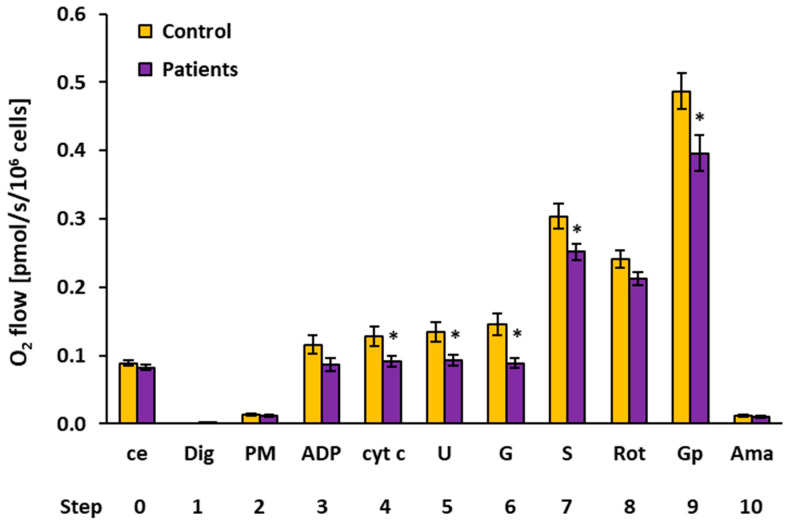
Platelet mitochondrial respiration by SUIT protocol 1 [17] measured in freshly isolated platelets expressed as O_2_ flow (pmol/s/10^6^ cells). A total of 200 × 10^6^ platelets were used in a 2 mL chamber of an O2k-respirometer. The respiration was measured in mitochondrial respiration medium MiR05 with 20 mM creatine at 37 °C under continuous stirring at 750 rpm. The columns show mean ± sem of the respiratory capacities after titration steps indicated on the x-axis. ce: intact cells; Dig: digitonin; PM: pyruvate plus malate; ADP: adenosine diphosphate; cyt c: cytochrome c; U: uncoupler; G: glutamate; S: succinate; Rot: rotenone; Gp: glycerophosphate; Ama: antimycin A. All substrates were titrated in kinetically saturating concentrations, and the uncoupler FCCP was titrated in optimum concentration to reach the maximum flux. The respiratory rates at step 9, Gp are 20–30% lower than the respiratory capacity at this state due to the use of lower than optimum uncoupler concentration at this titration step (see Section 4 for details). Control: the control group; Patients: the patients with UC. * *p* < 0.05—statistically significant difference vs. the control group.

**Figure 2:**
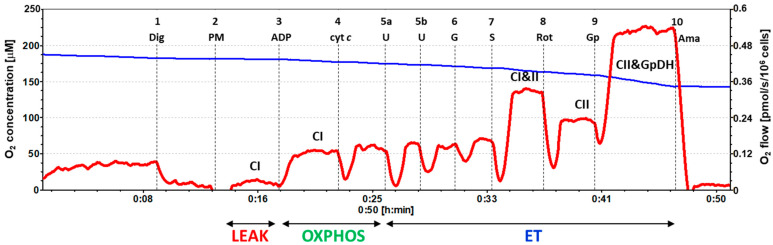
The trace from the measurement of platelet mitochondrial respiration in freshly isolated platelets following SUIT protocol 1. The blue line represents oxygen concentration (µM) and the red trace represents oxygen consumption as flow per cells (pmol O_2_/s/10^6^ cells). A total of 200 × 10^6^ platelets were used in a 2 mL chamber of an O2k-respirometer. The respiration was measured in mitochondrial respiration medium MiR05 with 20 mM creatine at 37 °C under continuous stirring at 750 rpm. The protocol includes the following titration steps: 1—digitonin (Dig); 2—pyruvate plus malate (PM); 3 —ADP; 4—cytochrome c (cyt *c*); 5a,b—uncoupler (U); 6—glutamate (G); 7—succinate (S); 8—rotenone (Rot); 9—glycerophosphate (Gp); 10—antimycin A (Ama). All substrates and inhibitors were added in saturating concentrations, and the uncoupler FCCP was titrated in optimum concentration to reach the maximum O_2_ flow at given respiratory state. The O_2_ flow after Step 9, Gp is 20–30% lower than the CII&GpDH-pathway respiratory capacity, as additional uncoupler titration is necessary to reach the maximum O_2_ flow at this state. The labels above the red trace indicate mitochondrial pathways involved in the respiratory rate: CI—complex I pathway; CI&II—complex I and complex II pathway; CII—complex II pathway; CII&GpDH—complex II and glycerophosphate dehydrogenase complex pathway; LEAK—non-phosphorylating resting state of respiration; OXPHOS—the phosphorylating state of respiration; ET—noncoupled state of respiration at optimum uncoupler concentration.

The results of the respirometric analysis of platelet mitochondrial respiration by the substrate–uncoupler–inhibitor titration (SUIT) protocol 1 are presented in Figure 1 and Table 2, showing respiratory capacities after indicated titration steps (see Section 4 and Figure 2 for details). Oxygen consumption in intact platelets (step 0) and CI-linked LEAK respiration (step 2) in patients with UC were similar to the control group. In UC patients, the CI-linked OXPHOS capacity (step 3, after ADP addition—the respiration associated with ATP production) was reduced to 74.5% of the control data and stayed lower (at 71.2% of control values, *p* = 0.044) after cytochrome c addition (step 4). CI-linked noncoupled respiration (CI-linked electron transfer (ET) capacity, step 5) was significantly lower in UC patients (at 69.3%, *p* = 0.023) vs. the control group. After CI-linked substrate glutamate addition (step 6), the ET capacity in the UC patients was significantly decreased vs. the control group (at 61.0%, *p* = 0.004). These results reflect a deficit in the CI-linked pathway in UC patients (see Figure 2). After adding complex II (CII)-linked substrate succinate (step 7), the respiration representing the ET capacity with CI&II-linked substrates was reduced in UC patients (at 82.8% of control values, *p* = 0.031). The CII-linked ET capacity (after rotenone addition, step 8) reached 88.2% of control values. The respiration after glycerophosphate addition (step 9) was significantly lower in UC patients (at 81.4% of control data, *p* = 0.020), indicating an impairment in the glycerophosphate pathway.

### 2.3. Platelet Mitochondrial Respiration and Fatty Acid Oxidation by SUIT Protocol 2

**Figure 3:**
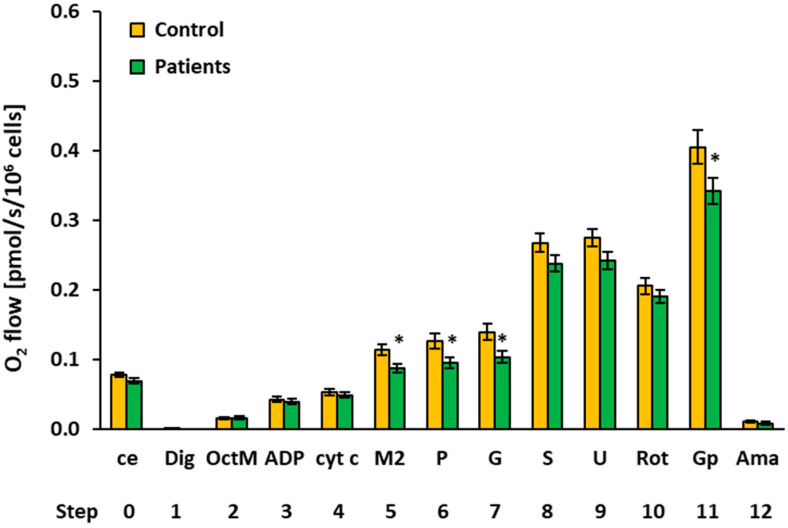
Platelet mitochondrial respiration by SUIT protocol 2 ([18], modified) measured in freshly isolated platelets expressed as O_2_ flow (pmol/s/10^6^ cells). A total of 200 × 10^6^ platelets were used in a 2 mL chamber of an O2k-respirometer. The respiration was measured in mitochondrial respiration medium MiR05 with 20 mM creatine at 37 °C under continuous stirring at 750 rpm. The columns show mean ± sem of the respiratory capacities after titration steps indicated on the x-axis. ce: intact cells; Dig: digitonin; OctM: octanoylcarnitine plus 0.1 mM malate saturating FAO; ADP: adenosine diphosphate; cyt c: cytochrome c; M2: 2 mM malate saturating I-linked respiration in the presence of pyruvate; p: pyruvate; G: glutamate; S: succinate; U: uncoupler; Rot: rotenone; Gp: glycerophosphate; Ama: antimycin A. All substrates were titrated in kinetically saturating concentrations, and the uncoupler FCCP was titrated in optimum concentration to reach the maximum O_2_ flow. The respiratory rates at step 11, Gp are 20–30% lower than the respiratory capacity at this state due to the use of lower than optimum uncoupler concentration at this titration step (for more details, see Section 4 ). Control: control group; Patients: the patients with urothelial carcinoma. * *p* < 0.05—statistically significant difference vs. the control group.

**Figure 4:**
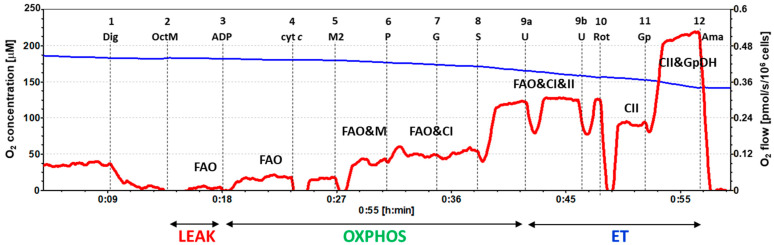
The trace from the measurement of platelet respiration in freshly isolated platelets following SUIT protocol 2. The blue line represents oxygen concentration (µM) and the red trace represents oxygen consumption as flow per cells (pmol O_2_/s/10^6^ cells). A total of 200 × 10^6^ platelets were used in a 2 mL chamber of an O2k-respirometer. The respiration was measured in mitochondrial respiration medium MiR05 with 20 mM creatine at 37 °C under continuous stirring at 750 rpm. The protocol includes following titration steps: 1—digitonin (Dig); 2—octanoylcarnitine plus 0.1 mM malate saturating FAO (OctM); 3—ADP; 4—cytochrome c (cyt *c*); 5—2 mM malate (M2) saturating CI-linked respiration in the presence of pyruvate; 6—pyruvate (P), 7—glutamate (G); 8—succinate (S); 9—uncoupler (U); 10—rotenone (Rot); 11—glycerophosphate (Gp); 12—antimycin A (Ama). All substrates and inhibitors were added in saturating concentrations, and the uncoupler FCCP was titrated in optimum concentration to reach the maximum O_2_ flow at given respiratory state. The O_2_ flow after Step 11, Gp is 20–30% lower than the CII&GpDH-pathway respiratory capacity, as additional uncoupler titration is necessary to reach the maximum O_2_ flow at this state. The labels above the red trace indicate mitochondrial pathways involved in the respiratory rate: FAO—fatty acid oxidation pathway; FAO&M—fatty acid oxidation and malate pathway; FAO&CI—fatty acid oxidation and complex I pathway; FAO&CI&II—fatty acid oxidation and complex I and complex II pathway; CII—complex II pathway; CII&GpDH—complex II and glycerophosphate dehydrogenase complex pathway; LEAK—non-phosphorylating resting state of respiration; OXPHOS—the phosphorylating state of respiration; ET—noncoupled state of respiration at optimum uncoupler concentration.

The results of platelet mitochondrial bioenergetics by SUIT protocol 2 are presented in Figure 3 and Table 3, showing respiratory capacities after indicated titration steps (see Section 4 and Figure 4 for details). Oxygen consumption in the intact platelets of patients with UC was similar to the control group (step 0). Fatty acid oxidation (FAO)-linked LEAK and OXPHOS respiration capacities (after the addition of FAO substrate octanoylcarnitine plus 0.1 mM malate and after ADP addition) (steps 2 and 3) were similar, as in the control group. However, platelet mitochondrial OXPHOS respiration was significantly decreased in patients with UC vs. the control group after 2 mM malate, pyruvate, and glutamate addition (steps 5, 6, and 7) (to 76.6%, *p* = 0.013, respectively, to 75.3%, *p* = 0.021 and to 74.0%, *p* = 0.021 of control group values). The difference was in the malate-anaplerotic-linked and CI-linked OXPHOS capacity (Figure 4). The deficit in the FAO&CI-linked OXPHOS capacity was compensated for by the addition of succinate, as no difference between groups was revealed in the FAO&CI&CII-linked OXPHOS capacity (step 8). There was no difference between groups in the FAO&CI&CII-linked ET capacity (step, 9), and the CII-linked ET capacity (step 10) was similar in both groups. The CII&GpDH-linked ET capacity (step 11) was lower in UC patients (at 84.4% of control group values, *p* = 0.049).

### 2.4. Flux Control Ratios of Platelet Mitochondrial Respiratory Capacities by SUIT Protocols 1 and 2

**Figure 5:**
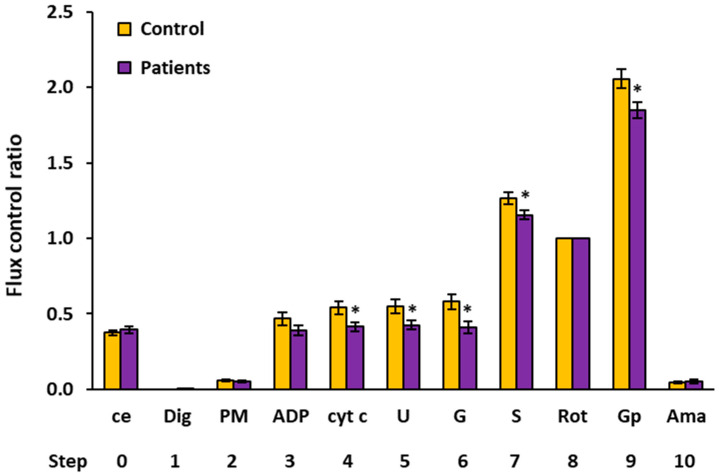
Flux control ratios of respiratory capacities measured in freshly isolated platelets by SUIT protocol 1. The CII-linked ET capacity (step 8) served as common reference state. The columns show mean ± sem of the FCR (relative units) after titration steps indicated on the x-axis. ce: intact cells; Dig: digitonin; PM: pyruvate plus malate; ADP: adenosine diphosphate; cyt c: cytochrome c; U: uncoupler; G: glutamate; S: succinate; Rot: rotenone; Gp: glycerophosphate; Ama: antimycin A. Control: the control group; Patients: the patients with UC. * *p* < 0.05—statistically significant difference vs. the control group. For more details, see Section 4 and the legend for Figure 1 and Figure 2 and Table 4.

**Figure 6:**
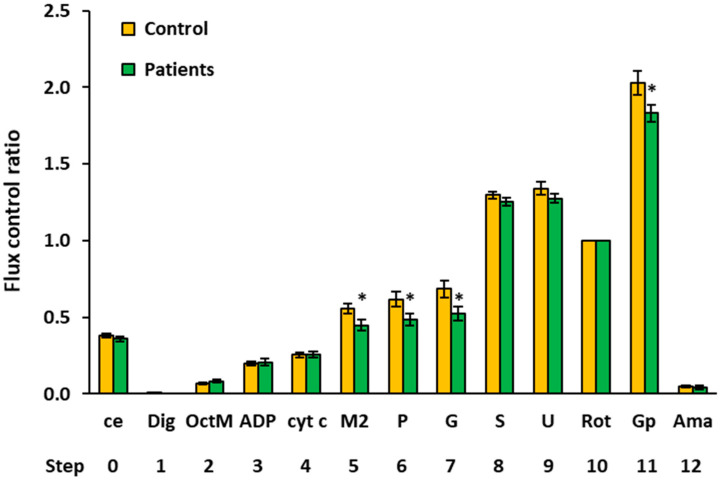
Flux control ratios of respiratory capacities measured in freshly isolated platelets by SUIT protocol 2. The CII-linked ET capacity (Step 10) served as common reference state. The columns show mean ± sem of the FCR (relative units) after titration steps indicated on the x-axis. ce: intact cells; Dig: digitonin; OctM: octanoylcarnitine plus 0.1 mM malate saturating FAO; ADP: adenosine diphosphate; cyt c: cytochrome c; M2: 2 mM malate saturating CI-linked respiration in the presence of pyruvate; P: pyruvate; G: glutamate; S: succinate; U: uncoupler; Rot: rotenone; Gp: glycerophosphate; Ama: antimycin A; Control: control group; Patients: the patients with UC. * *p* < 0.05—statistically significant difference vs. the control group. For more details, see Section 4 and the legend for Figure 3 and Figure 4 and Table 5.

As the CII-linked ET capacity did not differ between groups in either of the SUIT protocols, this respiratory rate (the mitochondrial respiration after addition of rotenone) was used in both protocols for the internal normalization of each measurement. The advantage of using ratios of fluxes is a higher statistical resolution of potential changes in the mitochondria quality that can be achieved, as the flux control ratios (FCR) are independent of the sample concentration [17,18]. The FCR of platelet mitochondrial respiratory capacities in control subjects vs. patients with UC by SUIT protocols 1 and 2 are shown in Figure 5 and Figure 6 and Table 4 and Table 5, respectively. The evaluation of FCR confirmed the deficit in CI-linked and malate-anaplerotic pathways, as the FCR of OXPHOS capacities with pyruvate plus malate (step 4, Figure 5), with octanoylcarnitine plus 2 mM malate (step 5, Figure 6), with OctPM (step 6, Figure 6), and with OctPGM (step 7, Figure 6) were lower in UC patients vs. the control group. The FCR of the ET capacity with succinate plus Gp was lower in UC patients vs. controls in both protocols (step 9, Figure 6 and step 11, Figure 2). These results show a relative deficit of CI-linked, malate-linked, and glycerophosphate dehydrogenase complex (CGpDH)-linked pathways in the platelet mitochondria of patients with UC in comparison to the control group.

### 2.5. Citrate Synthase Activity in Patients with Urothelial Carcinoma

A mitochondrial marker—the activity of citrate synthase (CS) in platelets—was decreased to 84.8% in patients with UC compared to controls (0.147 vs. 0.173 µmol/min/10^6^ cells, *p* = 0.0197). This may indicate a slightly decreased mitochondrial content in the platelets of patients with UC.

### 2.6. Endogenous Antioxidants and TBARS in Patients with Urothelial Carcinoma

The CoQ_10-TOTAL_ (ubiquinol plus ubiquinone) concentration in platelets was reduced to 75.5% of the control value (*p* = 0.0372), the concentration of α-tocopherol was decreased to 54.7% (*p* = 0.0200), and that of γ-tocopherol was decreased to 70.9% (*p* = 0.0019) of the control group value. The plasma concentration of β-carotene was significantly reduced to 58.7% (*p* = 0.0387) of the control group value. The parameter of oxidative stress, TBARS, was significantly increased in the plasma of UC patients (to 130.0% of control data, *p* = 0.0022). The endogenous concentration of CoQ_10-TOTAL_ (ubiquinone plus ubiquinol) in whole blood, as well as other measured antioxidants, did not differ between patients with UC and the control subjects (Table 6).

## 3. Discussion

Cancer is one of the major causes of death worldwide [19]. The early detection of cancer can help to improve therapy and enhance patient survival [20]. In the last years, platelet mitochondrial bioenergetics have been studied in various diseases [10,11,12,13,14,15,16], and also in thrombocytopenic patients undergoing chemotherapy [21]. In patients with ovarian cancer, alterations of the ultrastructure in platelets were detected [22]. The use of platelet characteristics is expected to provide an innovative strategy in the search for biomarkers of early-stage cancer [20].

Platelets are small circulating anucleate cell fragments generated from the megakaryocytes in the bone marrow. They are released from the bone marrow into circulation, where they live for 7–10 days [23]. Platelets are metabolically active and play an essential role in tumor growth and metastasis [22].

In this study, platelet counts were higher in UC patients compared to the controls. Among the causes of thrombocytosis is the capability of some tumor cells to produce thrombopoietin, and an upregulation of the platelet activation markers, such as P-selectin, β-thrombomodulin, or CD40 ligand, contributing to an increase in the platelet count [21]. Thrombocytes also display a pro-metastatic effect by producing platelet-derived TGF-β, which downregulates natural killer (NK) group 2 and member D (NKG2D), and results in the protection of tumor cells from NK cells. At the same time, epithelial-to-mesenchymal transition and tumor cell extravasation is promoted by the activation of TGF-β/Smad and NF-*κ*B signaling pathways [24]. In addition, TGF-β is partially responsible for the transformation of the neutrophils toward a pro-tumorigenic phenotype [25]. Preoperative and postoperative thrombocytosis was associated with worse outcomes in subjects with UC [26].

Platelets obtain approximately 60% of cellular energy by glycolysis and approximately 30–40% by OXPHOS [27]. Platelets have metabolic flexibility and the ability to utilize glycolysis instead of OXPHOS, which means that they can adapt to different situations for different diseases. Healthy platelets contain between five and eight mitochondria in a cell, which are critical for their function and survival [28].

The vital role of mitochondria in eukaryotic cells was demonstrated over a hundred years ago by Otto Warburg [29]. Mitochondria regulate important cellular processes, such as proliferation and cell death; they are the site of OXPHOS, the Krebs cycle, and FAO. Mitochondrial dysfunction participates in a wide spectrum of diseases, including cancer development. Several mechanisms could be included in cancer mitochondrial dysfunction, such as DNA mutations, increased reactive oxygen species (ROS) production, an acceleration of the opening of the voltage-dependent anion channel (VDAC), and the release of cytochrome c [30]. A significant deficit in the CI-linked OXPHOS and ET capacity in patients with UC is shown in Table 2. Our results agree with other studies, in which complex I dysfunction has been associated with cancer—in renal oncocytomas [31] and thyroid adenomas [32].

Our results highlight altered platelet mitochondrial functions in patients with urothelial carcinoma. The CI-linked OXPHOS and ET capacity were decreased in patients with UC vs. the control data. The deficit was also found in the malate anaplerotic pathway and glycerophosphate pathway. The malate anaplerotic pathway includes malic enzymes catalyzing the oxidative decarboxylation of L-malate to pyruvate. It is not clear from our experiment whether this pathway is downregulated, as the CI deficit could limit malate-linked respiration. Mitochondrial glycerophosphate dehydrogenase (mGpDH) is a key enzyme connecting OXPHOS, glycolysis, and fatty acid metabolism, and it is a site of high ROS production. Our results are in agreement with a previous study [32] that suggests that the platelet metabolism may be shifted from OXPHOS towards glycolysis in patients with UC. A deficient respiratory chain function and suppressed OXPHOS can induce anaplerotic pathways in platelet mitochondria. Our finding of significantly increased TBARS supports the presence of increased oxidative stress in patients with UC (Table 6).

Grasso et al. [30] introduced the definition “oncogenic mitochondria”—mitochondria that carry and can transfer malignant information. Cancer cells have different metabolic alterations, including the modification of the mitochondrial function, mutations of mitochondrial DNA (mtDNA), impairments of OXPHOS, a deficiency of antioxidants, and oxidative stress. However, the precise mitochondrial alterations in carcinogenesis allowing for anticancer interventions are not known.

The association between the circulating coenzyme Q_10_ and the risk of prostate cancer has been documented earlier [33]. Therefore, we suppose that the knowledge on the reduced platelet mitochondrial bioenergetic function and endogenous coenzyme Q_10_ level in patients with UC could improve targeting supplementary therapy with coenzyme Q_10_.

Possible mechanisms of the preventive actions and the possible application of different forms of vitamin E in various types of cancer were analyzed [34]. Recently, high concentrations of TBARS, a marker of oxidative stress, were found to be associated with poor survival in patients with metastatic UC [35].

Our current study found that the patients with UC have reduced CoQ_10_, γ-tocopherol, and α-tocopherol concentrations in platelets. We assume that this can contribute to the disorders of mitochondrial OXPHOS and the induction of oxidative stress. The patients with UC had significantly increased TBARS and decreased β-carotene plasma concentrations compared to the controls. The reduced CoQ_10_ concentration in platelets of the patients with UC to 75.5% vs. the control data indicates that this deficit could be limiting for electron transfer from complex I to complex III (Table 6). The organization of the respiratory complexes into supercomplexes with different interactions with the membrane lipid environment and CoQ pool [36] may explain that the deficit in electron transfer was not found in the CII-linked pathway in patients with UC. We suppose that decreased platelet endogenous CoQ_10_ biosynthesis can trigger the reverse electron transport from CoQ_10_ to complex I and can contribute to the reprogramming of platelet energy metabolism from mitochondrial OXPHOS to the activation of glycolysis. Altered CI-linked electron transfer activity to CoQ_10_ can increase ROS production and induce apoptosis, damage lipids, proteins, and mtDNA. Cytochrome c as a mobile part of the mitochondrial respiratory chain can be released through VDAC [37]. The mitochondrial function in platelets can reflect the mitochondrial health in the organism, but only in cancer cells could the cancer-specific metabolic changes be found. The reduction in complex II activity is associated with human cancer in renal carcinoma [38], and a higher activity of complex III has been detected in breast cancer [39].

To conclude, the observed changes in platelet mitochondrial bioenergetics are key for cell reprogramming in patients with UC. We suppose that increased oxidative stress, decreased OXPHOS, and a reduced platelet endogenous CoQ_10_ level can contribute to the reprogramming of platelet mitochondrial OXPHOS toward the activation of glycolysis. Furthermore, the impaired mitochondrial function can contribute to increased oxidative stress by initiating the reverse electron transport from CoQ_10_ to complex I. Our findings contribute to the new knowledge on the possible platelet role in the pathogenesis of UC.

## 4. Materials and Methods

### 4.1. Subjects

Control group: Fifteen healthy volunteers were enrolled in this study as the control group (6 men, 9 women), with a median age of 53 years (range 35–67 years). The inclusion criteria for healthy subjects were the following: no history of chronic disease including malignancy, absence of chronic medication, antithrombotic agents and contraceptives, and body mass index (BMI) < 26. Smoking, regular alcohol consumption, reduction diet, leukocytosis, lymphocytes > 40% of white blood cells (WBC), monocytes > 10% of WBC, and platelets > 350 × 10^6^/mL were exclusion criteria.

Patients with urothelial carcinoma: Fifteen consecutive chemotherapy-naïve patients (10 men, 5 women) with median age of 73 years (range 58–83 years) treated at the National Cancer Institute (NCI) in Bratislava, Slovakia between October 2020 and April 2021 were enrolled into this study. All patients had high-grade muscle-infiltrating urothelial carcinoma; one had plasmacytoid variant, and the other one had sarcomatoid differentiation. The primary site was bladder in 13 subjects and the upper genitourinary tract in 2 (renal pelvis in both cases). Two subjects had local disease (T2N0M0), seven with local disease (T3-4N0-3), and six patients displayed metastatic carcinoma (M1 disease). The baseline characteristics of patients are shown in Table 7. Inclusion criteria: histologically proven muscle-infiltrating urothelial carcinoma of the bladder or upper genitourinary tract. Exclusion criteria: prior systemic therapy, second primary malignancy except for basal cell skin carcinoma, and/or in situ cervical carcinoma in last five years.

After obtaining the approval of the Ethical Committee at the NCI in Bratislava, Slovakia (UC-SK001), and given written informed consent from all patients, pathologic, clinical, and radiologic data were entered by the physicians into electronic data files, and their accuracy was validated for each patient by an independent investigator. This study was carried out according to the Code of Ethics of the World Medical Association (Declaration of Helsinki) [40] and the principles of good clinical practice (GCP) [41].

### 4.2. Baseline Characteristics of the Groups

Patients with UC and control subjects did not differ in terms of body mass index (BMI), but differed in age (*p* < 0.0001) (Table 7). There were no significant differences reported in the parameters analyzed in this study between younger and older subjects, as well as between men and women; therefore, the different age and gender composition of the groups in this study did not represent potential confounding factors [42]. At a median follow-up of 2.8 months (range 0.6–6.0 months), 3 patients died due to disease progression.

### 4.3. Blood Count and Selected Metabolic Parameters of the Urothelial Carcinoma Patients and Control Subjects

The following parameters were measured in this study: white blood cells count, red blood cells count, platelets count, hemoglobin level, creatinine, urea, uric acid, γ-glutamyl transferase (GMT), alanine aminotransferase (ALT), aspartate transaminase (AST), alanine aminotransferase (ALP), total bilirubin, lipid parameters including triacylglycerols (TG), cholesterol, high-density lipoprotein (HDL) cholesterol, low-density lipoprotein (LDL) cholesterol, very low-density lipoprotein (VLDL) cholesterol, total protein, and albumin from peripheral blood plasma.

### 4.4. Antioxidants and Oxidative Stress Determination

Coenzyme Q_10-TOTAL_ (ubiquinol plus ubiquinone), α-tocopherol, γ-tocopherol, and β-carotene in whole blood, plasma, and isolated platelets were estimated using HPLC method with UV detection [43], modified by the authors [44,45]. TBARS, a measure of oxidative stress, was estimated by the spectrophotometric method [46].

### 4.5. Platelet Preparation

Blood samples were collected by venipuncture in two 9 mL K3EDTA (tripotassium ethylenediaminetetraacetic acid) tubes each day between 7:00 and 8:00 a.m. and transported to the laboratory at 25 °C. For platelet isolation, the tubes with blood were centrifuged at room temperature at 200× *g* for 10 min using a swing-out rotor without braking. Then, platelet-rich plasma (PRP) was transferred into a new plastic tube and mixed with 100 mM EGTA (ethylene glycol-bis(2-aminoethyl ether)-N, N, N’,N’-tetraacetic acid) to a final concentration of 10 mmol/L. The pellet, after centrifugation at 1200× *g*, was washed with 4 mL of DPBS (Dulbecco’s phosphate buffered saline) plus 10 mM EGTA and resuspended in 0.4 mL of the same solution. The platelet suspension was counted (10 times diluted) on hematological analyzer Mindray BC-6200 (Mindray, Shenzhen, China) [47].

### 4.6. High-Resolution Respirometry Method

Mitochondrial respiration was measured with high-resolution respirometry method [46,47]. For respirometric analysis, 200 × 10^6^ platelets were used in a 2 mL chamber of an O2k-respirometer (Oroboros Instruments, Austria). The respiration was measured in mitochondrial respiration medium MiR05 [48] with 20 mM creatine at 37 °C under continuous stirring at 750 rpm. Two different substrate–uncoupler–inhibitor titration (SUIT) protocols were applied [17,18,49] with common cross-linked respiratory states, allowing for harmonization of both protocols. The data were collected with DatLab software (Oroboros Instruments) using a data recording interval of 2 s [48]. All substrates and inhibitors were added in saturating concentrations, and the uncoupler was titrated in optimum concentration to reach the maximum O_2_ flow at given respiratory state. The O_2_ flow after Gp addition is 20–30% lower than the respiratory capacity, as additional uncoupler titration is necessary to reach the maximum O_2_ flow at this state.

#### 4.6.1. SUIT Protocol 1 for Evaluation of Platelet Mitochondrial Respiration

In the protocol 1 [17], after stabilization at routine respiration of intact cells, the substances were added in the following sequence: Step 1: digitonin (Dig) was added (0.20 µg/10^6^ cells) for cell permeabilization; Step 2: pyruvate (5 mM) and malate (2 mM) (PM) were added for determination of NADH-linked (CI-linked) LEAK respiration; Step 3: oxidative phosphorylation was stimulated by addition of 1 mM ADP-Mg (0.6 mol MgCl_2_/mol ADP) (ADP); Step 4: cytochrome c (10 µM) (cyt c) was added for testing the integrity of outer mitochondrial membrane; Step 5: stepwise titration of uncoupler FCCP (0.5 µM steps) (U) allowed for determination of noncoupled CI-linked respiration corresponding to electron transfer capacity (ET capacity) with CI-linked substrates; Step 6: addition of 10 mM glutamate (G) completed CI-linked pathway; Step 7: addition of 10 mM succinate (S) supported electron flow from CII-pathway into the Q-junction and allowed for determination of ET capacity of CI&II-pathway; Step 8: addition of CI inhibitor rotenone (1 µM) (Rot) inhibited electron flow from CI-pathway, allowing for determination of CII-linked ET capacity; Step 9: 10 mM glycerophosphate (Gp) was added to test the additional effect of CGpDH-pathway on ET-capacity (CII&GpDH-pathway); Step 10: addition of complex III (CIII) inhibitor antimycin A (2.5 µM) (Ama) inhibited mitochondrial respiration. As this respiration representing residual oxygen consumption was higher than the flux after the addition of digitonin, all respiratory fluxes were corrected for the flux after digitonin for evaluation of mitochondrial respiration. The representative trace of SUIT protocol 1 is presented in Figure 2.

#### 4.6.2. SUIT Protocol 2 for Evaluation of Platelet Mitochondrial Respiration and Fatty Acid Oxidation

The SUIT protocol 2 ([18], modified) was designed to give information on fatty acid oxidation (FAO)-pathway in OXPHOS state, avoiding FAO overestimation in the presence of anaplerotic pathways. In this protocol, after stabilization at routine respiration of intact cells, the substances were added in the following sequence: Step 1: digitonin (Dig) was added (0.20 µg/10^6^ cells) for cell permeabilization; Step 2: octanoylcarnitine (0.5 mM) and malate (0.1 mM) (OctM) were added (in concentrations saturating FAO) to induce fatty acid oxidation; Step 3: 1 mM ADP-Mg (0.6 mol MgCl_2_/mol ADP) (ADP) was added to stimulate OXPHOS with FAO-pathway substrates; Step 4: cytochrome c (10 µM) (cyt c) was added for testing the integrity of outer mitochondrial membrane. The increase in respiration after cytochrome c would indicate impaired integrity of outer mitochondrial membrane; Step 5: 2 mM malate (M2) was added to test for the presence of malic enzyme or other anaplerotic pathways (together FAO&M-pathway). Malate in 2 mM concentration is saturating for CI-linked respiration in the presence of pyruvate. The addition of 2 mM malate before pyruvate is the test for the presence of malic enzyme or other anaplerotic pathways by which pyruvate is formed from malate, and, therefore, malate stimulates CI-linked oxygen consumption similarly as in the presence of both pyruvate and malate; Step 6: 5 mM pyruvate (P) was added to support CI-pathway (together FAO&CI-pathway). As could be seen from the trace (Figure 6), relatively high activity of malic enzyme or other anaplerotic pathways is present in platelets, as addition of pyruvate after 2 mM malate causes only a small increase in respiration; Step 7: 10 mM glutamate (G) was added to complete CI-pathway; Step 8: 10 mM succinate (S) was added to support convergent electron flow from FAO&CI&II-pathway; Step 9: uncoupler FCCP was titrated to reach maximal ET capacity with FAO&CI&II-linked substrates; Step 10: rotenone (Rot) inhibited FAO and CI-linked respiration, allowing for determination of CII-linked ET capacity; Step 11: addition of 10 mM glycerophosphate (Gp) supported noncoupled respiration linked to CII&GpDH-pathways; Step 12: addition of 2.5 µM antimycin A (Ama) inhibited mitochondrial respiration at CIII. As this respiration representing residual oxygen consumption was higher than the flux after the addition of digitonin, all respiratory fluxes were corrected for the flux after digitonin for evaluation of mitochondrial respiration. The representative trace of SUIT protocol 2 is presented in Figure 4.

**Figure 7:**
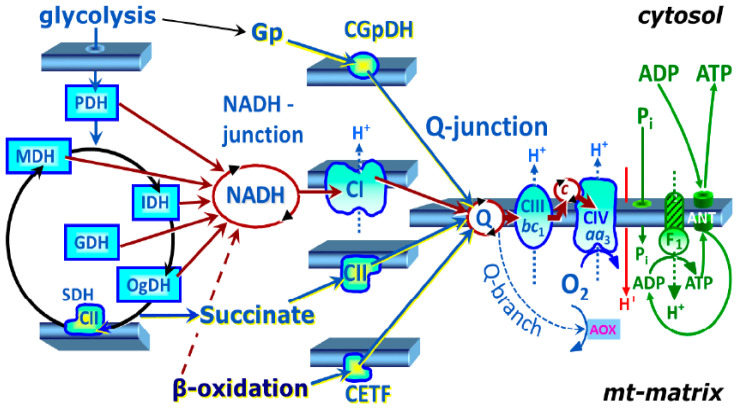
Mitochondrial electron transfer system—convergent electron transfer at the NADH-junction and Q-junction—© Gnaiger (2020), copied from [49] with permission. Electrons flow to oxygen from complex I (CI) or complex II (CII) and other flavoproteins, providing multiple entries into the Q-cycle (Q-junction). In the complete tricarboxylic acid (TCA) cycle in the living cell with the influx of pyruvate, electrons flow into Q-junction converges according to an NADH: succinate ratio of 4:1. Advanced SUIT protocols are designed for reconstitution of TCA cycle function and sequential separation of segments of mitochondrial pathways for OXPHOS analysis [48]. PDH—pyruvate dehydrogenase, MDH—malate dehydrogenase, IDH—isocitrate dehydrogenase, GDH—glutamate dehydrogenase, OgDH—2-oxoglutarate dehydrogenase, SDH—succinate dehydrogenase (CII), CETF—electron transfer flavoprotein complex, CGpDH—glycerophosphate dehydrogenase complex, Gp—glycerophosphate, ANT—adenine nucleotide translocase, AOX—alternative oxidase, bc_1_—the cytochrome b and cytochrome c_1_ of complex III (CIII), aa_3_—the cytochrome a and cytochrome a_3_ of cytochrome c oxidase (CIV), F_1_—the F_1_ subunit of ATP synthase attached to the transmembrane F_o_ subunit, Q—Q-cycle of coenzyme Q, mt-matrix—mitochondrial matrix.

The application of these two protocols allowed for comprehensive evaluation of mitochondrial pathways. The SUIT protocol 1 allowed for stepwise evaluation of CI-linked pathway followed by evaluation of CI&II-linked pathway, CII-linked pathway, and CII&GpDH-linked pathway. The SUIT protocol 2 allowed for evaluation of FAO-pathway followed by addition of malate-anaplerotic pathways (together, FAO&M-pathway), CI-linked pathway (together, FAO&CI-pathway), and CII-linked pathway (together, FAO&CI&II-linked pathway). Next, CII-linked pathway and CII&GpDH-linked pathway were evaluated. Schematic illustration of mitochondrial electron transfer system with dissected mitochondrial pathways is presented in Figure 7.

#### 4.6.3. Chemicals Used in SUIT Protocols for High-Resolution Respirometry

For high-resolution respirometry SUIT protocols, following chemicals (catalog number) were used: adenosine 5′-diphosphate sodium salt (A2754), antimycin A from Streptomyces sp. (A8674), L-glutamic acid monosodium salt hydrate (G1626), L(−)malic acid sodium salt (M1125), sodium pyruvate (P2256), sodium succinate dibasic hexahydrate (S2378), carbonyl cyanide 4-(trifluoromethoxy)phenylhydrazone (FCCP) (C2920) and rotenone (R8875) from Sigma-Aldrich, cytochrome c (24,804) from Merck, rac-glycerol 1-phosphate disodium salt hexahydrate (sc-215,789) from Santa Cruz Biotechnology, and ( ± )-octanoylcarnitine chloride (15,048) from Cayman.

### 4.7. Citrate Synthase Activity Determination

The activity of citrate synthase as mitochondrial marker was determined by spectrophotometric method, as described by [50].

### 4.8. Statistical Analysis

Data were summarized by frequency for categorical variables and by median and range for continuous variables. The differences between the groups were determined by log rank test. Differences were considered statistically significant when *p* < 0.05. All statistical analyses were performed using NCSS 20 Statistical Software, Kaysville, UT, USA [51].

## Figures and Tables

**Table 1:** Blood counts and selected metabolic parameters in patients vs. control subjects. * a difference is considered to be significant.

		N	Mean	Median	SD	SEM	*p*
Parameter (unit)							
White blood counts (10^9^/L)	Controls	15	6.23	5.90	1.87	1.09	<0.0930
	Patients	15	9.67	7.99	5.69	1.09	
Red blood counts (10^9^/L)	Controls	15	4.66	4.79	0.49	0.14	<0.0062 *
	Patients	15	4.04	3.83	0.60	0.14	
Platelet counts (10^9^/L)	Controls	15	247.47	243.00	64.69	43.93	<0.1710
	Patients	15	366.47	283.00	231.75	43.93	
Hemoglobin level (g/L)	Controls	15	140.67	139.00	13.30	4.08	0.0014 *
	Patients	15	117.07	110.00	17.97	4.08	
Creatinine (µmol/L)	Controls	15	74.12	75.10	12.92	7.58	<0.0002 *
	Patients	15	121.59	117.90	34.45	7.58	
Urea (mmol/L)	Controls	15	5.55	5.20	1.47	0.60	0.0003 *
	Patients	15	9.36	8.50	2.96	0.60	
Uric acid (µmol/L)	Controls	15	301.85	316.00	52.71	16.92	<0.0107 *
	Patients	15	378.94	371.00	72.53	16.92	
γ-glutamyl-transferase (GMT) (µkat/L)	Controls	14	0.38	0.31	0.22	0.08	<0.0107 *
	Patients	15	0.63	0.49	0.38	0.08	
Alanine aminotransferase (ALT) (µmol/L)	Controls	15	0.45	0.33	0.30	0.07	<0.5753
	Patients	15	0.40	0.32	0.23	0.07	
Aspartate transaminase (AST) (µmol/L)	Controls	15	0.41	0.36	0.14	0.04	<0.3718
	Patients	15	0.37	0.36	0.16	0.04	
Alanine aminotransferase (ALP) (µmol/L)	Controls	15	1.26	1.21	0.39	0.26	<0.2058
	Patients	15	1.82	1.58	1.36	0.26	
Total bilirubin (µmol/L)	Controls	15	13.67	11.60	6.58	1.47	<0.0326 *
	Patients	15	9.08	8.50	4.67	1.47	
Triacylglycerols (mmol/L)	Controls	15	2.05	1.21	1.97	0.39	<0.2627
	Patients	15	1.87	1.73	0.82	0.39	
Cholesterol (mmol/L)	Controls	15	5.32	5.53	1.06	0.29	<0.0326
	Patients	15	4.83	4.49	1.21	0.29	
High-density lipoprotein (HDL) cholesterol (mmol/L)	Controls	15	1.41	1.30	0.52	0.13	<0.7398
	Patients	15	1.30	1.34	0.44	0.13	
Low-density lipoprotein (LDL) cholesterol (mmol/L)	Controls	13	3.09	2.91	0.95	0.25	<0.2222
	Patients	15	2.68	2.30	0.87	0.23	
Very low-density lipoprotein (VLDL) cholesterol (mmol/L)	Controls	13	0.62	0.55	0.23	0.09	<0.0552
	Patients	15	0.85	0.79	0.37	0.08	
Total protein (g/L)	Controls	15	71.89	71.70	2.86	1.37	<0.0042 *
	Patients	15	65.34	64.50	6.91	1.37	
Albumin (g/L)	Controls	15	46.97	46.00	2.74	0.98	<0.0083 *
	Patients	15	42.53	42.00	4.62	0.98	

**Table 2:** Platelet mitochondrial respiration by SUIT protocol 1 [17] measured in freshly isolated platelets expressed as O_2_ flow (pmol/s/10^6^ cells). The table shows intact cell routine respiration and mitochondrial respiratory capacities after indicated titration steps in the protocol as mean, median, sd, sem. The *p*-values show statistical evaluation of differences between group of patients with UC and the control group. The mean respiratory capacities of UC patients are expressed as % of control group values. ce: intact cells; Dig: digitonin; PM: pyruvate plus malate; ADP: adenosine diphosphate; cyt c: cytochrome c; U: uncoupler; G: glutamate; S: succinate; Rot: rotenone; Gp: glycerophosphate; Ama: antimycin A. ^1^ The respiratory rates after addition of Gp are 20–30% lower than the respiratory capacity due to the use of lower than optimum uncoupler concentration at this titration step. * a difference is considered to be significant.

				O_2_ Flow (pmol/s/10^6^ Cells)					
Step	Titration		N	Mean	Median	Sd	Sem	*p*-Value	% of Control
0	ce	Controls	15	0.0885	0.0855	0.0160	0.0041		
		Patients	15	0.0820	0.0821	0.0156	0.0040	0.270	92.6%
1	Dig	Controls	15	0.0000	0.0000	0.0000	0.0000		
		Patients	15	0.0003	0.0000	0.0007	0.0002		
2	PM	Controls	13	0.0142	0.0113	0.0060	0.0017		
		Patients	14	0.0120	0.0110	0.0044	0.0012	0.298	84.9%
3	ADP	Controls	12	0.1160	0.1169	0.0477	0.0138		
		Patients	11	0.0865	0.0866	0.0322	0.0097	0.099	74.5%
4	cyt c	Controls	12	0.1285	0.1442	0.0500	0.0144		
		Patients	11	0.0916	0.0913	0.0288	0.0087	0.044 *	71.2%
5	U	Controls	12	0.1347	0.1473	0.0503	0.0145		
		Patients	11	0.0933	0.0962	0.0250	0.0075	0.023 *	69.3%
6	G	Controls	10	0.1456	0.1414	0.0522	0.0165		
		Patients	11	0.0888	0.0874	0.0242	0.0073	0.004 *	61.0%
7	S	Controls	15	0.3040	0.2803	0.0728	0.0188		
		Patients	13	0.2517	0.2547	0.0422	0.0117	0.031 *	82.8%
8	Rot	Controls	15	0.2408	0.2301	0.0488	0.0126		
		Patients	15	0.2124	0.2236	0.0378	0.0097	0.085	88.2%
9	Gp ^1^	Controls	15	0.4865	0.4509	0.1009	0.0260		
		Patients	15	0.3961	0.3735	0.1006	0.0260	0.020 *	81.4%
10	Ama	Controls	15	0.0113	0.0129	0.0060	0.0016		
		Patients	15	0.0102	0.0101	0.0079	0.0020	0.679	90.5%

**Table 3:** Platelet mitochondrial respiration by SUIT protocol 2 ([18], modified) measured in freshly isolated platelets expressed as O_2_ flow (pmol/s/10^6^ cells). The table shows intact cell routine respiration and mitochondrial respiratory capacities after indicated titration steps in the protocol as mean, median, sd, sem. The *p*-values show statistical evaluation of differences between group of patients with UC and the control group. The mean respiratory capacities of UC patients are expressed as % of control group. ce: intact cells; Dig: digitonin; OctM: octanoylcarnitine plus malate; ADP: adenosine diphosphate; cyt c: cytochrome c; M2: malate; P: pyruvate; G: glutamate; S: succinate; U: uncoupler; Rot: rotenone; Gp: glycerophosphate; Ama: antimycin A. ^1^ The respiratory rates after addition of Gp are 20–30% lower than the respiratory capacity due to the use of lower than optimum uncoupler concentration at this titration step. * a difference is considered to be significant.

				O_2_ Flow (pmol/s/10^6^ Cells)					
Step	Titration		N	Mean	Median	Sd	Sem	*p*-Value	% of Control
0	ce	Controls	15	0.0775	0.0797	0.0116	0.0030		
		Patients	13	0.0689	0.0702	0.0117	0.0032	0.062	88.9%
1	Dig	Controls	15	0.0004	0.0000	0.0014	0.0004		
		Patients	14	0.0000	0.0000	0.0000	0.0000	0.343	
2	OctM	Controls	15	0.0145	0.0131	0.0071	0.0018		
		Patients	12	0.0155	0.0153	0.0063	0.0018	0.718	106.5%
3	ADP	Controls	15	0.0421	0.0426	0.0150	0.0039		
		Patients	13	0.0390	0.0380	0.0142	0.0039	0.584	92.7%
4	cyt c	Controls	15	0.0528	0.0563	0.0184	0.0047		
		Patients	14	0.0481	0.0493	0.0148	0.0040	0.463	91.2%
5	M2	Controls	11	0.1129	0.1183	0.0261	0.0079		
		Patients	14	0.0865	0.0929	0.0230	0.0062	0.013*	76.6%
6	P	Controls	12	0.1257	0.1382	0.0360	0.0104		
		Patients	14	0.0947	0.0990	0.0278	0.0074	0.021 *	75.3%
7	G	Controls	12	0.1392	0.1440	0.0423	0.0122		
		Patients	14	0.1030	0.1049	0.0334	0.0089	0.023 *	74.0%
8	S	Controls	15	0.2671	0.2535	0.0517	0.0134		
		Patients	14	0.2374	0.2308	0.0446	0.0119	0.110	88.9%
9	U	Controls	15	0.2744	0.2703	0.0489	0.0126		
		Patients	14	0.2418	0.2370	0.0474	0.0127	0.080	88.1%
10	Rot	Controls	11	0.2055	0.2044	0.0389	0.0117		
		Patients	14	0.1899	0.1792	0.0352	0.0094	0.304	92.4%
11	Gp ^1^	Controls	11	0.4047	0.3827	0.0811	0.0245		
		Patients	13	0.3415	0.3155	0.0677	0.0188	0.049 *	84.4%
12	Ama	Controls	13	0.0107	0.0116	0.0065	0.0018		
		Patients	14	0.0079	0.0048	0.0076	0.0020	0.303	73.2%

**Table 4:** Flux control ratios for respiratory capacities measured in freshly isolated platelets by SUIT protocol 1. The CII-linked ET capacity (Step 8) served as common reference state. The table shows FCR (relative units) for intact cell routine respiration and mitochondrial respiratory capacities after indicated titration steps in the protocol as mean, median, sd, sem. The *p*-values show statistical evaluation of differences between group of patients with UC and the control group. The mean FCR of respiratory capacities of UC patients are expressed as % of control group. ce: intact cells; Dig: digitonin; PM: pyruvate plus malate; ADP: adenosine diphosphate; cyt c: cytochrome c; U: uncoupler; G: glutamate; S: succinate; Rot: rotenone; Gp: glycerophosphate; Ama: antimycin A. ^1^ The FCR of the respiratory rates after addition Gp are 20–30% lower than the FCR of respiratory capacity due to the use of lower than optimum uncoupler concentration at this titration step. * a difference is considered to be significant.

				Flux Control Ratio (Relative Units)					
Step	Titration		N	Mean	Median	Sd	Sem	*p*-Value	% of Control
0	ce	Controls	15	0.3741	0.3668	0.0721	0.0186		
		Patients	15	0.3934	0.3767	0.0880	0.0227	0.517	105.1%
1	Dig	Controls	15	0.0000	0.0000	0.0000	0.0000		
		Patients	15	0.0014	0.0000	0.0037	0.0010	0.156	
2	PM	Controls	13	0.0574	0.0517	0.0197	0.0055		
		Patients	11	0.0513	0.0500	0.0158	0.0048	0.423	89.5%
3	ADP	Controls	12	0.4665	0.4773	0.1465	0.0423		
		Patients	11	0.3894	0.3963	0.1140	0.0344	0.176	83.5%
4	cyt c	Controls	11	0.5393	0.5868	0.1359	0.0410		
		Patients	11	0.4141	0.4453	0.1057	0.0319	0.026 *	76.8%
5	U	Controls	12	0.5466	0.5699	0.1620	0.0468		
		Patients	11	0.4250	0.4602	0.0998	0.0301	0.044 *	77.8%
6	G	Controls	10	0.5799	0.5639	0.1507	0.0476		
		Patients	11	0.4115	0.3682	0.1285	0.0388	0.012 *	71.0%
7	S	Controls	15	1.2635	1.2688	0.1537	0.0397		
		Patients	13	1.1550	1.1352	0.1136	0.0315	0.046 *	91.4%
8	Rot	Controls	15	1.0000	1.0000	0.0000	0.0000		
		Patients	15	1.0000	1.0000	0.0000	0.0000		
9	Gp ^1^	Controls	14	2.0569	2.0941	0.2302	0.0615		
		Patients	15	1.8476	1.9119	0.2053	0.0530	0.015 *	89.8%
10	Ama	Controls	15	0.0474	0.0439	0.0266	0.0069		
		Patients	15	0.0505	0.0398	0.0445	0.0115	0.822	106.4%

**Table 5:** Flux control ratios for respiratory capacities measured in freshly isolated platelets by SUIT protocol 2. The CII-linked ET capacity (Step 10) served as common reference state. The table shows FCR (relative units) for intact cell routine respiration and mitochondrial respiratory capacities after indicated titration steps in the protocol as mean, median, sd, sem. The *p*-values show statistical evaluation of differences between group of patients with UC and the control group. The mean FCR of respiratory capacities of UC patients are expressed as % of control group. ce: intact cells; Dig: digitonin; OctM: octanoylcarnitine plus malate; ADP: adenosine diphosphate; cyt c: cytochrome c; M2: malate; P: pyruvate; G: glutamate; S: succinate; U: uncoupler; Rot: rotenone; Gp: glycerophosphate; Ama: antimycin A. ^1^ The FCR of respiratory rates after addition of Gp are 20–30% lower than the FCR of respiratory capacity due to the use of lower than optimum uncoupler concentration at this titration step. * a difference is considered to be significant.

				Flux Control Ratio (Relative Units)					
Step	Titration		N	Mean	Median	Sd	Sem	*p*-Value	% of Control
0	ce	Controls	15	0.3811	0.3867	0.0546	0.0141		
		Patients	14	0.3584	0.3484	0.0589	0.0157	0.291	94.0%
1	Dig	Controls	15	0.0019	0.0000	0.0075	0.0019		
		Patients	14	0.0000	0.0000	0.0000	0.0000	0.343	
2	OctM	Controls	15	0.0695	0.0668	0.0302	0.0078		
		Patients	12	0.0832	0.0772	0.0371	0.0107	0.300	119.7%
3	ADP	Controls	15	0.2015	0.2119	0.0511	0.0132		
		Patients	13	0.2082	0.2114	0.0782	0.0217	0.788	103.3%
4	cyt c	Controls	15	0.2539	0.2439	0.0673	0.0174		
		Patients	14	0.2565	0.2579	0.0763	0.0204	0.925	101.0%
5	M2	Controls	11	0.5558	0.5781	0.1084	0.0327		
		Patients	13	0.4479	0.4264	0.1303	0.0361	0.040 *	80.6%
6	P	Controls	12	0.6185	0.6716	0.1590	0.0459		
		Patients	13	0.4873	0.4853	0.1380	0.0383	0.037 *	78.8%
7	G	Controls	12	0.6844	0.7221	0.1882	0.0543		
		Patients	13	0.5247	0.5347	0.1593	0.0442	0.031 *	76.7%
8	S	Controls	15	1.2968	1.3176	0.0847	0.0219		
		Patients	14	1.2522	1.2478	0.1025	0.0274	0.211	96.6%
9	U	Controls	15	1.3416	1.3182	0.1591	0.0411		
		Patients	14	1.2753	1.2335	0.1169	0.0313	0.215	95.1%
10	Rot	Controls	15	1.0000	1.0000	0.0000	0.0000		
		Patients	14	1.0000	1.0000	0.0000	0.0000		
11	Gp ^1^	Controls	10	2.0291	2.0845	0.2561	0.0810		
		Patients	13	1.8305	1.8184	0.1913	0.0531	0.045 *	90.2%
12	Ama	Controls	15	0.0496	0.0516	0.0305	0.0079		
		Patients	14	0.0427	0.0271	0.0416	0.0111	0.612	86.1%

**Table 6:** Endogenous antioxidants and TBARS in patients with UC. TBARS: thiobarbituric acid reactive substances. * a difference is considered to be significant.

		N	Mean	Median	Sd	Sem	*p*-Value	% of Control
Whole blood (µmol/L)								
Coenzyme Q_10-TOTAL_	Controls	14	0.313	0.321	0.077	0.026		
	Patients	15	0.333	0.351	0.111	0.025	0.5126	106.4%
α-tocopherol	Controls	15	20.82	20.20	6.481	1.549		
	Patients	15	23.33	22.60	5.477	1.549	0.1198	112.1%
γ-tocopherol	Controls	15	1.387	1.400	0.926	0.186		
	Patients	15	1.184	1.260	0.426	0.186	0.8195	85.4%
β-carotene	Controls	14	0.249	0.249	0.171	0.042		
	Patients	15	0.144	0.144	0.142	0.040	0.0701	57.8%
Platelets (pmol/10^9^ cells)								
Coenzyme Q_10-TOTAL_	Controls	12	84.14	79.5	19.26	5.39		
	Patients	14	63.56	69.65	18.14	4.99	0.0372 *	75.5%
α-tocopherol	Controls	13	2546.5	2091.9	1482.0	321.1		
	Patients	15	1392.9	1174.9	778.5	298.9	0.0200 *	54.7%
γ-tocopherol	Controls	14	325.6	292.2	157.9	72.3		
	Patients	15	231.0	106.0	343.2	69.8	0.0019 *	71.0%
Plasma (µmol/L)								
Coenzyme Q_10-TOTAL_	Controls	13	0.516	0.498	0.114	0.037		
	Patients	15	0.466	0.462	0.15	0.035	0.3449	90.3%
α-tocopherol	Controls	15	31.14	31.40	8.78	1.946		
	Patients	15	31.40	32.30	6.04	1.946	0.3614	100.8%
γ-tocopherol	Controls	15	2.086	1.46	1.352	0.263		
	Patients	15	1.542	1.55	0.498	0.263	0.4806	73.9%
β-carotene	Controls	14	0.370	0.291	0.223	0.050		
	Patients	14	0.217	0.152	0.143	0.050	0.0387 *	58.6%
TBARS	Controls	14	5.035	4.905	0.829	0.301		
	Patients	15	6.545	6.160	1.342	0.290	0.0022 *	130.0%

**Table 7:** Baseline characteristics of groups. N: number of patients; BMI: body mass index; ECOG: Eastern Cooperative Oncology Group; TNM: tumor, node, metastasis; * each patient might have more than one distant metastasis; ** both pleural and lenticular metastases in one patient.

		Median (Range)	N (%)	*p*
Age (years)	Control subjects	53 (35–67)	15 (100)	
	UC patients	73 (58–83)	15 (100)	<0.0001
Male/female	Control subjects		6/9 (40.0/60.0)	
	UC patients		10/5 (66.7/33.3)	
BMI (kg/m^2^)	Control subjects	25.5 (17.9–32.9)		
	UC patients	25.2 (17.2–40.4)		0.885
UC patients				
Primary tumor site	Bladder/renal pelvis		13/2 (86.7/13.3)	
Histology type	Urothelial carcinoma		15 (100.0)	
Histology variants	Plasmacytoid/sarcomatoid		1/1 (6.7/6.7)	
ECOG performance status	≤1		10 (66.7)	
	>1		5 (33.3)	
TNM classification	Local disease (T2N0M0)		2 (13.3)	
	Locally advanced disease (T3-4N0-3M0)		7 (46.7)	
	Metastatic disease (M1)		6 (40.0)	
Metastasis localization *	Distant lymph nodes		2 (33.3)	
	Bones		3 (50.0)	
	Lungs		4 (66.7)	
	Liver		2 (33.3)	
	Peritoneum		2 (33.3)	
	Other **		1 (16.7)	

## Data Availability

The data presented in this study are available on request from the corresponding author. The data are not publicly available due to privacy.

## Abbreviations

Ama	Antimycin A
BMI	Body mass index
ce	Intact cells
CI	Complex I
CII	Complex II
CIII	Complex III
CoQ_10_	Coenzyme Q_10_
CoQ_10-TOTAL_	Ubiquinol plus ubiquinone
cyt c	Cytochrome c
Dig	Digitonin
ET	Electron transfer
FAO	Fatty acid oxidation
FCCP	Carbonyl cyanide p-trifluoro-methoxyphenyl hydrazone
G	Glutamate
Gp	Glycerophosphate
M2	Malate 2 mM
N	Number of patients
NADH	Nicotinamide adenine dinucleotide reduced
OctM	Octanoylcarnitine plus malate 0.1 mM
OXPHOS	Oxidative phosphorylation
P	Pyruvate
PDGF	Platelet-derived growth factor
PM	Pyruvate plus malate
Rot	Rotenone
S	Succinate
SUIT	Substrate–uncoupler–inhibitor titration
TBARS	Thiobarbituric acid reactive substances
TGF-β	Transforming growth factor β
U	Uncoupler
VEGF	Vascular endothelial growth factor
